# Supplementary material for: Splice-Junction-Based Mapping of Alternative Isoforms in the Human Proteome
Source: Cell Rep. Author manuscript; Available in PMC 2020 Jan 15. (PMC6961840; doi:10.1016/j.celrep.2019.11.026)
Supplement: 3 [file NIHMS1546469-supplement-3.zip › DF2/PXD009737/TestisLysC-96-Q9P0W8-SNAAVDCSVPVSVSTSIK.pdf]

A

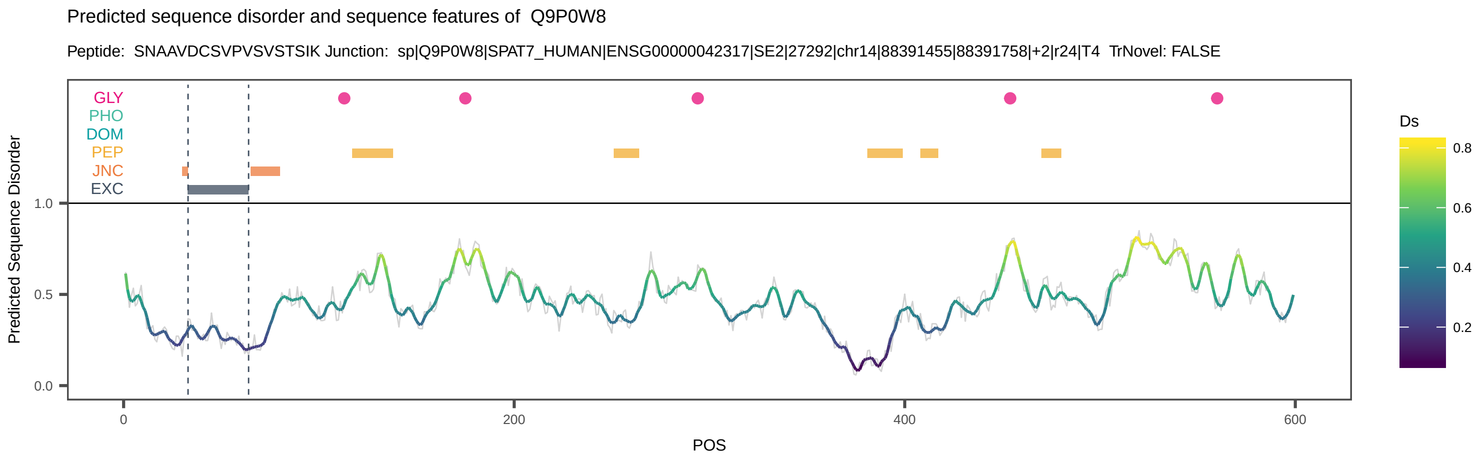

Predicted sequence disorder and sequence features of Q9P0W8
Peptide: SNAAVDCSVPVSVSTSIK Junction: sp|Q9P0W8|SPAT7_HUMAN|ENSG00000042317|SE2|27292|chr14|88391455|88391758|+2|r24|T4 TrNovel: FALSE
GLY
PHO
DOM
PEP
JNC
EXC
Predicted Sequence Disorder
1.0
0.5
0.0
0
200
400
600
POS
Ds
0.8
0.6
0.4
0.2

B

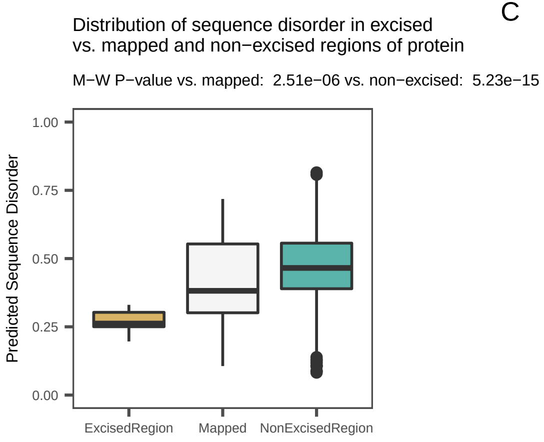

Distribution of sequence disorder in excised
vs. mapped and non−excised regions of protein
M−W P−value vs. mapped: 2.51e−06 vs. non−excised: 5.23e−15
Predicted Sequence Disorder
1.00
0.75
0.50
0.25
0.00
ExcisedRegion
Mapped
NonExcisedRegion

C
